# A Randomized Clinical Trial of the Acceptability and Preliminary Effectiveness of an Online CBT Platform in Diverse Youth

**DOI:** 10.3390/brainsci16080853

**Published:** 2026-08-13

**Authors:** Margaret Danielle Weiss, Rajendra Aldis, Danta D. R. Bien-Aime, Taylor Witkowski, Peyton Williams, Katie E. Holmes, Dharma E. Cortés, Philip S. Wang, Benjamin Lê Cook, Eleanor Castine Richards

**Affiliations:** 1 Cambridge Health Alliance, 1493 Cambridge St., Cambridge, MA 02139, USA; raaldis@challiance.org (R.A.); dbienaime@challiance.org (D.D.R.B.-A.); twitkowski@challiance.org (T.W.); pewilliams@challiance.org (P.W.); decortes@challiance.org (D.E.C.); bcook@cha.harvard.edu (B.L.C.); erichards@challiance.org (E.C.R.); 2 Department of Psychiatry, Harvard Medical School, Boston, MA 02215, USA; pswang@mgb.org

**Keywords:** digital mental health interventions, cognitive behavioral therapy, youth mental health disparities

## Abstract

**Background**: The objective of this study was to evaluate the preliminary effectiveness of a self-administered digital mental health intervention, COPE2Thrive, in improving symptoms and functional impairment in youth in a diverse community setting. **Methods**: Outcome measures included self-reported Kiddie-Computerized Adaptive Testing (K-CAT) scores on seven diagnostic domains, and self-reported scores of six domains and the total score of the Weiss Functional Impairment Rating Scale (WFIRS)—Self Report. All youth were eligible to participate unless they were currently in another therapy. Primary analyses followed the stepped-wedge randomized design, comparing participants assigned to one of three randomized intervention sequences. Secondary analyses treated the study as an open-label intervention study and evaluated pre- to post-treatment change among participants who engaged in the intervention, using the last observation carried forward for non-completers. **Results**: We successfully recruited a majority non-White sample that was 31.5% Black, 31.5% Hispanic/Latino, and 19.6% Asian. Of the eligible participants, 30.5% enrolled in the study, 23.4% completed the intervention, and 12.5% participated in the follow-up. Racial and ethnic minority subjects were less likely to have received outside behavioral health therapies, both in the screening sample and among those who participated in the intervention. There was no statistically significant improvement in the total score or any of the domain scores on the K-CAT or the WFIRS-S, either in the RCT or in the open-label analysis. **Conclusions**: Given the low acceptability of the program and the absence of clinically significant improvement in this pilot study, the findings suggest that the intervention, as implemented, may have limited utility for addressing youth distress in diverse communities.

## 1. Introduction

The CDC Youth Mental Health Survey conducted in 2021 described persistent feelings of sadness and hopelessness in 57% of youth, with the greatest levels of distress in adolescents, particularly girls in their late teens. A mixed-methods study using an online survey of 593 young people found that during the COVID-19 pandemic, 48% had clinical depression, 51% had anxiety, and both clinical and non-clinical samples reported loneliness [1,2]. The Surgeon General, Vivek Murthy, in partnership with the American Academy of Pediatrics and the American Academy of Child and Adolescent Psychiatry, issued a joint declaration of a national emergency in youth mental health. The advisory recommended “research to identify and respond to youth mental health needs more rapidly”, especially the “needs of at-risk youth.” Interventions to address the youth mental health crisis (YMHC) would have to be scalable, low-cost, and youth-friendly. Such an intervention could, it was hoped, be delivered in school settings at minimal cost, as well as in diverse communities [3,4]. This option was of particular interest to those who care for diverse youth as a potential avenue to decrease clear disparities in access to care for minoritized youth [5,6].

Digital mental health interventions (DMHIs) are not dependent on clinicians and can, therefore, circumvent barriers posed by a shortage of providers. DMHIs can be delivered anonymously and, thus, destigmatize care. DMHIs can be adapted for delivery—either to targeted populations with an identified disorder or universally in a community setting. Another significant advantage of DMHIs is that they can be delivered in rural areas geographically isolated from large mental health centers, which may be one reason that DMHIs have been of particular interest in countries where service to remote areas is essential, such as Canada [7], Finland [8], and Australia [9,10]. DMHIs have also been considered as possible early interventions in adolescence to prevent progression to chronic serious mental health illness in adulthood. DMHIs can be integrated into a stepped care approach as early interventions designed to build resilience prior to the onset of serious mental illness.

There are now a plethora of apps and web platforms focused on mental health, most of which lack empirical testing [11]. In addition, more research is looking at the acceptability, retention, and effectiveness of digital interventions among racially and ethnically diverse youth, which is an important target population given the wide and persistent disparities in mental health care access. A majority of DMHI platforms are adapted from various evidence-based psychological treatments, but without validation of the digital adaptation.

A recent systematic review looked at 45 RCTs of online mental health interventions for youth [12], of which fourteen of the RCTs used web-based self-help platforms to deliver cognitive behavioral therapy (CBT). Eighty-two percent of the studies were tested in high school or college students. The authors report that, of the interventions that were empirically tested, 64% were found to be effective. Two independent reviewers reviewed and scored the quality of the studies using the Critical Appraisal Checklist for RCTs. Based on these scores, 10/13 studies were considered “good”; 7–9 were “moderate”; and <7 were “poor”. The nature of the limitations described may favor findings of greater effectiveness than would have been identified using a more rigorous methodology. Methodological limitations included a lack of blinding, selection bias, failure to report baseline differences, inclusion of extra contacts that were not part of the intervention, failure to perform intent-to-treat analyses, and an insufficient description of lost participants. RCTs that were found to be ineffective included a problem-solving intervention [13]; a cluster randomized trial of an e-coach anxiety program [14]; and an RCT of acceptance and commitment therapy to promote adolescent mental health [15]. These results suggest that the digital adaptation of well-established and evidence-based face-to-face interventions requires empirical validation in their own right, even when adapted from an evidence-based, face-to-face intervention.

A systematic review of DMHIs for youth has identified the strengths or limitations of DMHI research; the methodological limitations have been described above. Retention in DMHIs was as low as 28%, which led some of the studies to include additional interpersonal contacts that were not part of the program being tested to drive sufficient recruitment to even be able to complete them. The review concluded that low youth engagement was a key limitation of web-based online therapies and, as a result, some authors suggest that it is essential to evaluate the acceptability and feasibility of an intervention prior to scaling up implementation to large populations [12,16]. Study characteristics associated with a greater likelihood of effectiveness include using CBT, specifically targeting populations with internalizing difficulties, and using internalizing symptoms as the outcome. Broad outcomes, such as quality of life, functional impairment, or coping behavior, were more resistant to change. Studies using passive rather than active controls, and those including an interpersonal component, were also more likely to be effective.

Poor retention rates suggest the need for more research to improve acceptability, especially since the acceptability of an intervention is significantly correlated to its effectiveness [16]. A study of adolescent perceptions of online therapy found that 72% of adolescents said they would access online therapy if they were experiencing mental health problems, but only 32.9% would choose online therapy over face-to-face support [17], confirming that acceptability remains a key limitation for DMHIs. In summary, these findings highlight the importance of measuring the acceptability and retention of DMHIs, using intent-to-treat (ITT) approaches, and evaluating both symptom-specific outcomes and broad-based outcomes of real-world functional impairment, quality of life, coping, and treatment satisfaction.

DMHIs have, on occasion, been tested or adapted for some of the specific populations that use them, such as racial and ethnic minority groups [18], disadvantaged and underserved youth [19], homeless youth [20], and LGBTQ youth [21]. Studies of the sociodemographic variables that are associated with acceptability are needed to determine which therapies will be most appropriate for whom, and for what outcomes [22]. A systematic review of DMHIs for adolescents with mental health conditions in low-resource settings found 18 systematic reviews and meta-analyses, but none of the studies reported sample-specific metrics, even though they were being applied in low-resource settings with marginalized groups [11]. Given that there is a clear disparity in access to mental health care for racial and ethnic minority adolescents [23], success in using DMHIs to address the needs of these youth requires that they be evaluated in diverse, real-world community settings.

There are two recent studies of DMHIs delivered in schools in which the methodology met most of the methodological standards described above. The first study was a pilot of a co-designed app that established the feasibility of a school-hosted frontline mental health promotion and prevention initiative for adolescents using transdiagnostic symptom outcomes—as measured by the Strengths and Difficulties Questionnaire—and a universal rather than targeted approach [24]. The second study was a large RCT (*N* = 275) of a DMHI (coping camp) to reduce stress in Chinese high school students, which was sufficiently powered to detect even small effect sizes [25]. This was an unblinded, cluster RCT comparing 275 subjects who received the DMHI to 265 students receiving treatment as usual. Outcome measures looked at stress, depression, anxiety, stress-coping behaviors, and mental health well-being. On average, students completed 8.5 out of 11 sessions, suggesting that the duration of treatment had low acceptability. The study found a very small effect size of 0.1 for perceived stress, anxiety, and depression, but no significant effect on any of the real-world outcomes, including stress-coping behaviors, functional outcome, or mental health well-being. While the authors report the study as finding the DMHI to be effective on the basis of statistically significant improvements in symptoms based on being well-powered to find minor effects, the clinical significance of the observed changes is questionable. An improvement with an effect size of 0.1 for specific symptoms represents a change lower than the MCID of the measure, with no improvement in function, coping, or well-being, which suggests that the intervention had minimal real-world clinical impact.

In the study reported here, we evaluated a DMHI that was adapted from Creating Opportunities for Personal Empowerment (COPE) Health Lifestyles TEEN (Thinking, Emotions, Exercise, and Nutrition). COPE TEEN is a 15-week face-to-face CBT program that has been found to have good feasibility, acceptability, and outcomes in an RCT of 779 high school students using an attention control [26,27]. The seven-session digital COPE2Thrive program for adolescents was subsequently developed as a self-administered online intervention (www.cope2thrive.com). This is the first empirical study of the acceptability, feasibility, and effectiveness of the seven-session digital intervention.

This RCT was part of a larger initiative on Early Screening and Treatment of at-Risk Youth (ESToRY), which was a research initiative focused on improving mental health care for children, adolescents, and young adults under 25 in racial, ethnic, and language (REL)-minority communities. Two hundred and nineteen high school students within the Greater Boston area were screened in ESToRY [28]. About a third of this non-clinical sample met the criteria for clinical depression or anxiety, and fifty percent met the criteria for two or more DSM-5 disorders of moderate severity along with functional impairment greater than 1.5 standard deviations outside the norm. This C2T study was a mixed-methods study. We report the quantitative results here; the qualitative results will be reported separately.

## 2. Materials and Methods

### 2.1. Recruitment

Flyers containing a QR code for interested participants were posted in high schools and community-based centers throughout the Greater Boston area. Research assistants were present in schools, classrooms, school-based health centers, and at community events to provide information on the study and recruit eligible youth. Participation and recruitment were hybrid: either all or any of the study procedures could be conducted in person or virtually. All youth assented, and parents consented if the youth was younger than 18 years. Trial registration: This study was registered with ClinicalTrials.Gov on 3 January 2022 (NCT04935710); the trial protocol and statistical analysis plan can be accessed at https://clinicaltrials.gov/study/NCT04935710?term=cope2thrive&viewType=Card&rank=1#more-information (accessed on 1 August 2026). 

#### 2.1.1. Eligibility Criteria

Youth aged 12–24 years were eligible to participate in the C2T intervention if they were high school students enrolled in one of the schools within the study’s catchment area; were fluent in English, Haitian Creole, Portuguese, or Spanish; and had completed the baseline screening assessments of the parent ESToRY study. Youth were excluded if they were currently receiving therapy; were in 12th grade and, therefore, unlikely to be available for the duration of the study follow-up period; or were identified as actively suicidal and in need of urgent clinical care at the time of screening. A total of 128 of the 219 screened youth met the eligibility criteria. Of these, 40 provided consent or assent; however, one youth who consented to the study did not complete the baseline C2T survey. Therefore, 39 participants enrolled and initiated the C2T program, 30 completed the intervention, and 16 completed the three-month follow-up assessment. This corresponded to an enrollment rate of 30.5% among eligible youth, an intervention–completion rate of 23.4%, and a three-month follow-up rate of 12.5%.

#### 2.1.2. Patient and Public Involvement

Recruitment materials and study procedures were developed by study staff in collaboration with schools, a community advisory board consisting of youth/young adults and parents/caregivers of youth with lived experience, and other community organization staff to ensure they were attractive and appropriate for youth participants. Participants were not involved in the statistical analysis.

### 2.2. Outcome Measures

#### 2.2.1. Symptoms and Diagnoses

The Kiddie-Computerized Assessment Test (K-CAT) [26] provides a rating of mild, moderate, or severe based on a score out of 100 for seven domains: depression, anxiety, Attention Deficit Hyperactivity Disorder (ADHD), Oppositional Defiant Disorder (ODD), Conduct Disorder, Mania, Substance Use, and Suicide Risk. This evaluation draws on a bank of 2120 items and uses an adaptive algorithm to select the most relevant items for the participant as they enter their responses. The primary outcome was the total K-CAT score across all domains.

The K-CAT was previously validated in 801 North American youth, which included but were not exclusive to diverse populations. The K-CAT modules were tested for predictive, discriminant, and convergent validity as compared to the gold standard of the Kiddie Schedule for Affective Disorders and Schizophrenia, which has AUCs between 0.83 and 0.99 [29]. The K-CAT was then specifically tested in two different high-risk samples of youth, looking at suicide and substance use. The K-CAT was also linguistically and culturally validated in Spanish and is designed to provide accurate cross-cultural validity for Spanish speakers.

#### 2.2.2. Functional Impairment

Youth self-reported how their emotional and behavioral well-being was impacting their school learning and behavior, self-concept, high-risk behaviors, family, social engagement, and life skills with the Weiss Functional Impairment Rating Scale—Self Report (WFIRS-S) [28]. Each item is scored from (0) not at all to (3) very much. Items scored as not applicable do not contribute to the mean score. Functional outcome was analyzed by looking at both the mean score for each domain and the overall mean WFIRS score.

The WFIRS-S was previously validated in an adolescent school sample, as well as in a wide range of other settings and ethnic groups. The population norms for age and gender used for this study were collected in a sample of 3130 youth in North America, of which 651 scored in the clinical range. T scores < 60 were classified as normal; T scores between 60 and 65 were classified as at risk; and T scores > 65 were classified as impaired (unpublished manual). The WFIRS-S was previously translated into 27 languages, and validation studies performed in Canada, the US, France, Turkey, Japan, Spain, Germany, and Iran have shown similar findings, suggesting consistent psychometric properties independent of ethnic or cultural diversity. These studies confirmed acceptable or strong psychometric properties, including test–retest reliability, internal consistency, cross-informant reliability, and convergent and divergent validity. Given its reliability in multiple cultures and in low- and middle-income countries, the WFIRS-S was considered to demonstrate sufficient reliability and cultural validity for this community study of diverse youth.

#### 2.2.3. Risk Score

An algorithm was developed to integrate symptom (K-CAT) and functional (WFIRS) outcomes into a single clinical risk score as normal (Tier 1), at risk (Tier 2), or clinically ill (Tier 3). Tier 1 included youth who were either functioning in the normal range, even if they were symptomatic, or youth who were asymptomatic but had some functional impairment. Tier 2 included youth with moderate or severe symptoms and functional impairment above 1.5 standard deviations of the norm. Tier 3 included youth with two or more moderate or severe diagnoses associated with significant functional impairment. Twenty-nine percent of the youth in Tier 3 were in clinical treatment.

The evaluation of symptoms, functioning, and risk score was performed at treatment baseline, end of treatment, and at the 3-month follow-up.

#### 2.2.4. Screening and Follow-Up Procedures

Youth who completed the baseline screening were given immediate feedback on their scores by the research assistants. The feedback interview opened by pointing out areas of strength, followed by discussion of the areas that the youth had described as challenging, and then asking open-ended questions such as, “Do these results describe how you see things?” Research assistants received ongoing training in interrater reliability and qualitative responsiveness to youth, employing role-play exercises and supervision to assure youth-friendly language. The brief motivational interview that followed screening was intended to motivate participation in the digital intervention where appropriate. The C2T program was presented as a possible strategy to support coping skills.

Adverse events were systematically assessed throughout the study period through the K-CAT assessments, and participants identified as high risk were referred for immediate clinical evaluation according to the study protocol. No serious adverse events related to the intervention were reported.

#### 2.2.5. Randomization to the C2T Intervention: Stepped-Wedge Design

To assess the preliminary effectiveness of the C2T intervention, we used a pre-registered three-group stepped-wedge design, with all participants in the treatment as usual (TAU) group at baseline, and then with groups randomly “crossed over” from TAU into the treatment group at different time periods. The study was originally powered to enroll 108 youth. To account for anticipated loss to follow-up and high rate of study decline, a total of 128 youth were ultimately randomized. Participants were assigned to one of three sequences, with crossover timing modified from monthly intervals (as originally planned) to weekly intervals to decrease loss to follow-up: group 1 (*n* = 43), group 2 (*n* = 43), or group 3 (*n* = 42). Group 1 received 1 week of treatment as usual (TAU) prior to initiating the COPE2Thrive (C2T) intervention; group 2 received 2 weeks of TAU prior to initiating the C2T intervention; and group 3 received 3 weeks of TAU before initiating the intervention. The random allocation sequence was generated by study staff. Allocation was implemented using a central, password-protected spreadsheet that contained the randomization sequence. The study staff responsible for enrollment assigned participants to the next available sequence according to the pre-specified randomization order after completion of screening and enrollment. Only the designated study staff responsible for participant assignment had access to the allocation sequence. Due to the nature of the stepped-wedge design, participants and study staff were not blinded to the intervention assignment. 

### 2.3. Statistical Analyses

**Figure 1:**
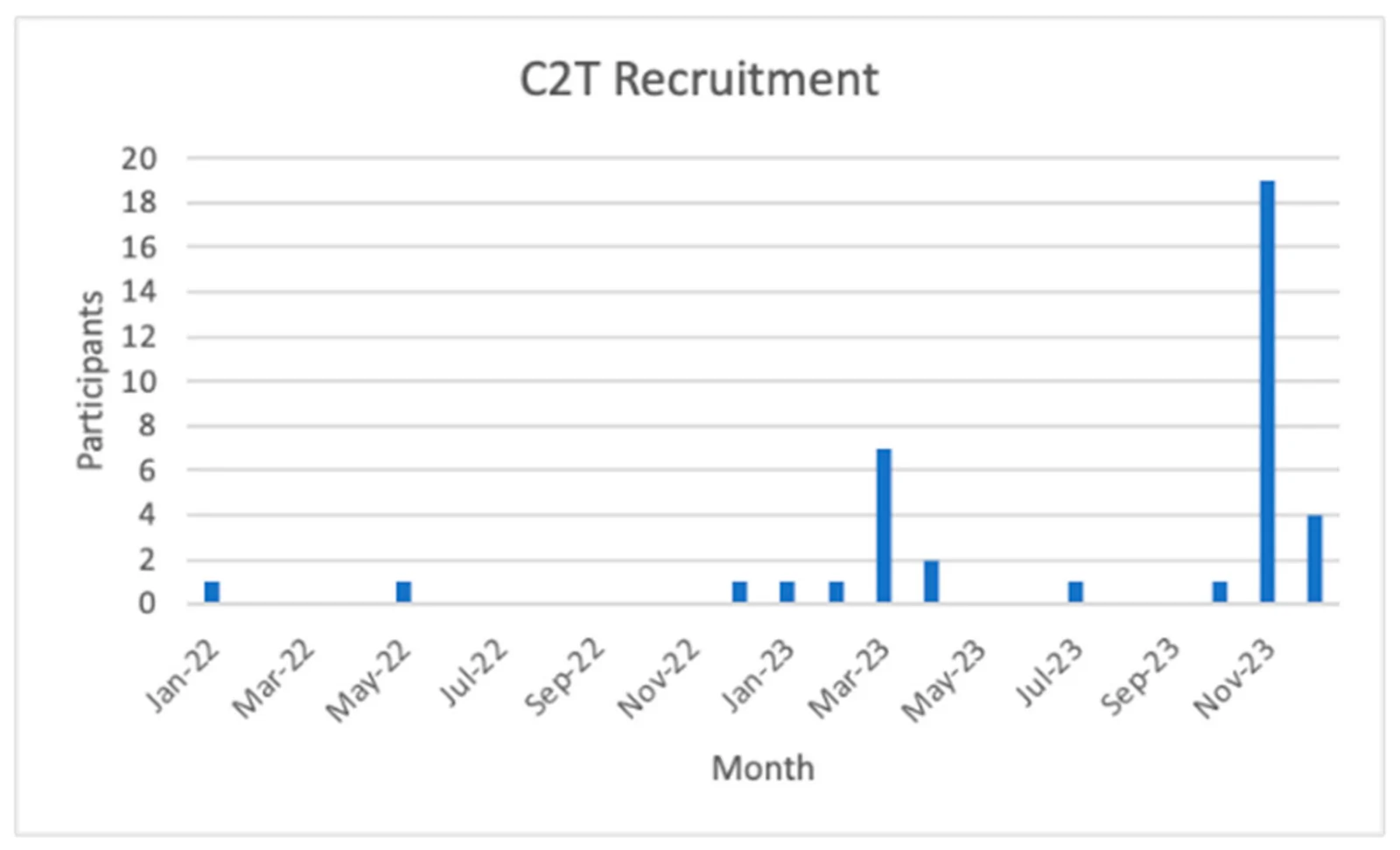
Timeline for C2T recruitment.

We first described the demographics (age, race and ethnicity, and language spoken at home) of the screened population (*n* = 219). Next, we assessed variation in the demographics among those that did and did not agree to participate in the C2T intervention among the eligible population (*n* = 128). Chi-square tests were used to test the statistical significance of group differences (Table 1). The timing of the C2T recruitment is presented in Figure 1.

In accordance with the pre-registered analysis of the randomized controlled trial, we conducted an adjusted regression analysis, following an intent-to-treat (ITT) framework in which participants were analyzed according to their original stepped-wedge randomization assignment, regardless of the timing of intervention initiation. Intervention exposure was modeled as a time-varying indicator reflecting assignment to the C2T program versus treatment as usual (TAU), according to the pre-specified stepped-wedge schedule. Models included fixed effects for the study period—specified as categorical time indicators with the first period as the reference—to control for secular trends over time; additionally, a random intercept for participants was included to account for within-individual correlation across repeated measurements. To address heterogeneity in the timing of intervention initiation due to delayed crossover, models were additionally adjusted for time since initial screening. Robust (sandwich) standard errors were used to account for small sample size and potential model misspecification. Missing outcome data were handled using multiple imputation with multivariate normal (MVN) imputation, generating 10 imputed datasets (see Appendix A for the ITT results).

Given the null findings from the pre-registered ITT analyses, we conducted a post hoc, exploratory, per-protocol analysis of the 39 subjects that initiated treatment with the C2T program. We measured the unadjusted changes in symptom, functional-impairment, and clinical-tier outcomes from baseline to completion (Table 2). We next measured the changes from baseline to three-month follow-up in the diagnostic symptom and functional outcomes among those who completed the three-month follow-up assessment (Table 3).

## 3. Results

### 3.1. Demographics of the Youth Screened and Offered the C2T Intervention

The study population was drawn from public high schools in racially, ethnically, and linguistically diverse communities. The total screening sample (*n* = 219) ranged in age from 12 to 24 years. Of these, 70.8% were female, 21.9% were male, 4.1% were non-binary, and 3.1% preferred not to answer. The screening population was able to self-identify and select multiple racial/ethnic categories. In turn, the participants were 31.5% Black; 31.5% were Latino/Hispanic; 19.6% were Asian; 38.8% were White; and 7.3% were “other”. Almost half the youth (48.8%) described their ethnicity as American, while the remainder identified as African American (15.5%); Hispanic (23.7%); Asian (16.0%); or Brazilian, Haitian, Jamaican, or other. All subjects spoke English, but 47% of subjects spoke another language at home. Overall, 79% of the sample came from racial, ethnic, or language minority backgrounds. These demographics demonstrate an over-recruitment of minoritized youth, both when compared to the community and when compared to the demographics of their respective schools [30].

Of the 219 youth screened, 56 (25.5%) were excluded because they were in active therapy. Racial, ethnic, and language minority youth were significantly (X2(3, *N* = 219) = 15.53, *p* = 0.001) less likely to be in therapy (60.66% of White participants in therapy versus 18.03% of Black/African American, 34.43% of Hispanic/Latino, and 16.39% of Asian participants). As a result, non-White youth were more likely to be eligible for the C2T intervention because they were less likely to be excluded due to currently receiving therapy.

### 3.2. Acceptability

The proportion of youth who agreed to participate out of all those who were eligible was used as a quantifiable approximation of acceptability. Previous studies have shown a correlation between participation, completion, and intervention acceptability [31], suggesting that low participation is a reasonable proxy for poor acceptability. A detailed qualitative review of the youth report on the acceptability of the intervention will be included in the qualitative report of this mixed-methods study. Youth declined for a variety of reasons, such as concerns about schoolwork and time, and that they did not want to have to watch videos. A prominent theme, elaborated upon in detail in the qualitative study, was that youth felt isolated and lonely following online schooling and the pandemic and were looking for interpersonal connection as opposed to “online therapy”.

Of the 128 deemed eligible for the C2T intervention, 39 agreed to participate. The 39 participants were not equally distributed across the stepped-wedge groups (group 1, *n* = 10; group 2, *n* = 16; group 3, *n* = 13). Of these, 22 participants initiated the intervention according to their assigned stepped-wedge sequence (group 1 (*n* = 5), group 2 (*n* = 9), and group 3 (*n* = 8)). The remaining 17 participants initiated the intervention later than scheduled, with delays ranging from 60 to 775 days after the initial screening (group 1 (*n* = 5), group 2 (*n* = 7), and group 3 (*n* = 5)).

Participants who agreed to enroll in the C2T program were significantly less likely to be Hispanic/Latino (12.8%), more likely to identify as Asian (25.6%), more likely to speak English at home (59%), and less likely to speak Spanish at home (2.6%) (Table 1). Although minoritized subjects were more likely to be eligible for the C2T program, the recruited sample remained disproportionately White relative to the overall screened population.

Recruitment for the screening phase of the study was conducted from January 2022 through February 2024. Follow-up assessments were completed through March 2024. Of the 41 subjects approached in Year 1 to participate, only one subject agreed to participate, and that subject did not complete the intervention. The trial was completed as planned but enrolled fewer participants than originally anticipated (*n* = 39 vs. 108 anticipated). Figure 1 illustrates the recruitment over time, with the increase in recruitment likely driven by protocol amendments to increase the incentive for participation from $35.00 to $100.00 and by expanding the inclusion criteria to include all three tiers as long as they were not in treatment.

As outlined, protocol amendments over the course of the project were implemented to boost recruitment. On 7 August 2023, eligibility was expanded to include youth from all three tiers, and, on 19 September 2023, the incentive to participate was increased from $35 USD to $100 USD. The majority of subjects were recruited in the last 3 months of the study in response to increasing the incentive.

### 3.3. Intent-to-Treat: Stepped-Wedge Analysis

Adjusted regression analyses using multiply imputed data and mixed-effects regression models consistent with the stepped-wedge design showed no statistically significant effects of the COPE2Thrive (C2T) intervention on the K-CAT outcome (β = 0.86, 95% CI −4.53 to 6.24, *p* = 0.37) or on any other outcome. Across all models, assignment to the C2T program, relative to treatment as usual, was not associated with significant improvement in outcomes, even after adjustments for the study period, time since initial screening, and accounting for within-participant correlation. Estimates were consistent in direction and magnitude across imputed datasets, and conclusions were unchanged when accounting for delayed crossover and secular time trends (see Appendix A). Adjusted regression analyses yielded wide confidence intervals, which indicate a lack of statistical precision attributable to the constrained sample size. These stepped-wedge findings, when considered in isolation, are insufficient to definitively preclude the potential effectiveness of the C2T intervention.

### 3.4. Per-Protocol Analyses: Changes in Symptoms and Functional Impairment

Analysis of changes from pre- to post-treatment in subjects who participated did not demonstrate any statistically significant improvements. There was no improvement in any of the seven diagnostic symptom groups and suicidality, the seven domains of functional impairment, or the overall score (Table 2). The data show very modest numerical improvement in some diagnoses and domains of functional impairment (Table 2 and Table 3), none of which were statistically significant. Given the low completion rate (*n* = 30) and three-month follow-up (*n* = 16) for the per-protocol sample, confidence intervals are wide and reflect the low statistical precision associated with our limited sample size.

We conducted a post hoc sensitivity power analysis for the per-protocol post hoc analysis and found that, with an alpha of 0.05 and a desired power of 0.80, we were powered to detect an effect size of Cohen’s d = 0.46. We were not powered to find a small effect size.

### 3.5. Change in Tier Assignment

An initial aim of the study protocol was to determine if early intervention in an at-risk population of youth would decrease the number of youth at risk, who are therefore vulnerable to becoming seriously mentally ill in adulthood. The pandemic changed the context of this objective, in that the study ran during the youth mental health crisis, with almost half of youth already ill (Tier 3), and 25% more at risk (Tier 2). Only a quarter of the community youth were not clinically or functionally impaired. No statistically significant change in tiers was observed, but we did see a decrease in youth who were classified as Tier 3 (10 vs. 5) and a slight increase in the number of youth classified as Tier 2 (Table 2). These numbers are too small for statistical analysis. The protocol was written pre-pandemic in the hope of determining whether the digital intervention could build coping skills that would prevent youth at risk (Tier 2) from going on to develop serious mental illness (Tier 3). Instead, as a result of the very high rate of morbidity found in youth during the mental health crisis, what we observed was a small shift from Tier 3 (clinically ill) to Tier 2 (at risk).

### 3.6. Adverse Events

No serious adverse events or unintended harms related to the intervention were reported during the study period.

## 4. Discussion

The implementation of the stepped-wedge design deviated from the original protocol to reduce loss to follow-up (i.e., the timing of crossover to the treatment group was compressed to one week, and several participants experienced delays in intervention initiation). To compensate, we estimated mixed-effects regression models, adjusting for both secular time trends and individual time since initial screening to disentangle the treatment effect from the temporal delays. However, these protocol modifications limit the causal inferences that can be drawn from the stepped-wedge estimates. Even if our mixed-effects models controlled for both secular trends and individual time since screening to isolate the impact of the C2T program from these delays, such modifications necessarily constrain the causal conclusions that can be derived from the stepped-wedge results.

Recruitment was low and only began to pick up after we increased the incentive. The sample is, therefore, likely to be subject to systematic bias in favor of participants who were more engaged, more motivated, or possibly more responsive to monetary study incentives. Even in this biased sample, we did not find any clinically or statistically significant responses.

Using participation as a quantitative measure of acceptability provides a narrow and potentially misleading perspective on true acceptability. In this study, participation would introduce systematic bias in favor of overestimating acceptability, since participation was negligible until the incentive was substantially increased towards the end of the study. Real-life acceptability where subjects would have paid for the program, rather than being paid to use the program, would have been substantially lower. The qualitative study will elaborate on more specific attitudes towards the intervention, both from those who refused and those who participated.

This study had 19 outcome measures (eight symptom domains, eight functional domains, and three tier scores). Although we might have anticipated improvements in some variables by chance alone, no improvement was found in any of the outcome variables.

A strength of this study was our success in recruiting a diverse sample. The focus of this study was on racial, ethnic, and language minority youth considered to be most at risk. Minoritized youth are frequently underrepresented in mental health research, despite experiencing high levels of stress and adversity [32]. Several aspects of the study design likely contributed to this success. There was symmetry between the race and ethnicity of our research staff and the youth we recruited. We worked closely with an advisory board that had close roots with the communities we were working with. Use of virtual and/or in-person access to research procedures may have mitigated many of the usual barriers to participation. As a result of our success in recruiting a minoritized sample, we were able to replicate previous findings in our screening sample, demonstrating that minoritized youth are less likely to participate in behavioral health interventions, and, further, that this disparity in access for minority populations was equally true for the digital intervention. The participants who ultimately engaged in the digital mental health intervention were statistically more likely to be White.

Digital mental health interventions (DMHIs) typically elicit substantial non-specific and placebo-mediated responses—frequently characterized as “digital placebos”—whereby the mere provision of clinical attention or self-monitoring tools can reduce perceived stress and enhance mood, regardless of therapeutic core content. The failure to find placebo improvement is striking, and is consistent with our other findings in suggesting that youth were reluctant to embrace a self-directed, web-based platform as therapeutic.

Our study findings are remarkably consistent with the results found in the coping camp study, with the exception that the coping camp study was well-powered, and, thus, able to identify a very small effect size for improvements in depression and anxiety, which we would have missed [25]. Even with this level of power, the coping camp study failed to find improvements in perceived stress, mental health well-being, or coping behaviors. An effect size (ES) of 0.1 may have been statistically significant but would not be considered clinically significant. The Minimal Clinically Important Difference (MCID) for the Depression Anxiety Stress Scale is 5 points, and the study found a change of 2.69 points [33]. In a study with very high power, a very low effect size that does not represent a perceptible change in well-being is not a positive finding. Furthermore, this very small change was transitory and disappeared at the follow-up. These very small benefits in symptoms would have minimal real-world impact at the community level.

This study further supports prior research findings that low acceptability is highly correlated to poor outcome, and that DMHI studies should consider testing acceptability prior to launching an RCT [16]. DMHI interventions have the unique advantages of being scalable, inexpensive, and easily accessible. On the other hand, our study and other empirical studies of DMHIs demonstrate that digital interventions may also suffer from a combination of low desirability, low efficacy, and lack of effectiveness in meaningful outcomes, such as in functional impairment, coping, well-being, or stress. Our study was carried out at the heart of the “shadow pandemic”, in which youth were returning to in-person schooling after being isolated from their peers during the pandemic. Our results suggest that offering a digital intervention to treat digital fatigue, loneliness, and social isolation may have been perceived by youth as adding insult to injury.

Our study was able to demonstrate that, despite multiple sources of Type I error leading to bias in demonstrating a false-positive effect, our results consistently show no improvement in any of the 19 outcome measures at any point in time. While we were underpowered to show improvements with a small ES, given that acceptability was so low, small improvements in a program that youth were not inclined to use would not be expected to show real-world benefits.

## 5. Conclusions

Although the C2T program is a scalable, inexpensive intervention based on evidence-based therapy and shown to be effective in person [27,34], our findings suggest that this DMHI has significant limitations. An intervention that lacks both acceptability and effectiveness is unlikely to provide benefit at the community level, especially among diverse youth most at risk. In conclusion, despite the advantages of digital interventions, even DMHIs adapted from in-person treatments that are known to be effective need to be empirically tested prior to being promoted for profit, for clinical use, or as a community-level intervention to address the youth mental health crisis.

## Figures and Tables

**Table 1:** Demographics of C2T participants vs. non-participants (%).

	Participated in C2T (*n* = 39)	Declined (*n* = 89)	*p* < 0.05
Race/Ethnicity			
White	30.08 (18.3, 46.9)	18.0 (11.3, 27.5)	
Black	28.2 (16.3, 44.3)	28.1 (19.7, 38.4)	
Latino	12.8 (5.4, 27.5)	36.0 (26.6, 46.5)	*
Asian	25.6 (14.3, 41.6)	14.6 (8.6, 23.7)	*
Other	2.6 (0.3, 16.3)	3.4 (1.1, 10.0)	
Gender			
Female	69.2	67.4	
Male	28.2	24.7	
Non-binary	0	3.4	
Prefer not to answer	2.6	4.5	
Age	15.7 (15.3, 16.1)	15.9 (15.6, 16.2)	
Language at Home			
English	59.0 (43.0, 73.2)	49.4 (391., 59.8)	*
Haitian Creole	7.7 (2.5, 21.5)	5.6 (2.3, 12.9)	
Portuguese	7.7 (2.5, 21.5)	10.1 (5.3, 18.4)	
Spanish	2.6 (3.5, 16.3)	19.1 (12.2, 28.7)	*
Other	23.1 (12.4, 38.9)	15.7 (9.5, 24.9)	

* Statistically significant at the *p* < 0.05 level.

**Table 2:** Changes in symptoms, function, and risk tier from baseline to completion (*n* = 30).

Symptom Domain	Baseline	Completion
Anxiety	42.9 (20.8, 71.0)	41.4 (10.0, 62.6)
Mania	35.5 (6.7, 59.4)	29.5 (0.9, 63.3)
ODD	29.9 (0.8, 62.2)	27.4 (0.4, 52.1)
ADHD	30.8 (6.2, 62.9)	31.2 (1.1, 58.5)
Depression	41.8 (15.8, 73.7)	38.5 (4.6, 70.8)
Conduct	19.5 (0.9, 57.7)	14.8 (0.9, 70.1)
Suicide risk	37.0 (11.7, 73.3)	32.5 (0.3, 66.4)
SUD	35.7 (4.8, 75.7)	33.0 (4.8, 66.4)
Functional Domain		
Family	0.35 (0, 1.1)	0.32 (0, 2.0)
Work	0.37 (0, 1.2)	0.32 (0, 2.0)
School	0.48 (0, 1.3)	0.50 (0, 1.3)
Life	0.54 (0, 1.7)	0.51 (0, 1.3)
Concept	0.99 (0, 2.4)	0.84 (0, 3.0)
Social	0.27 (0, 1.4)	0.17 (0, 0.7)
Risk	0.08 (0, 0.5)	0.05 (0, 0.5)
Overall	0.44 (0, 1.1)	0.39 (0, 1.1)
Number in Each Risk Tier		
Tier 1	11	10
Tier 2	12	10
Tier 3	10	5
Tier 3—high risk	4	3
Missing *	2	2
Total	39	30

No results were statistically significant at the *p* < 0.05 level. * Missing due to incomplete K-CAT or WFIRS data—clinical tier assignment required complete K-CAT and WFIRS data.

**Table 3:** Changes in symptoms, function, and risk tier from baseline to 3-month follow-up (*n* = 16).

Symptom Domain	Baseline	Follow-Up
Anxiety	41.4 (23.3, 64.9)	32.6 (6.8, 52.4)
Mania	33.8 (6.7, 52.0)	24.8 (0.2, 54.7)
ODD	28.3 (0.8, 48.2)	22.1 (0.7, 44.3)
ADHD	26.4 (6.2, 48.5)	21.1 (5.3, 47.7)
Depression	38.8 (15.8, 61.6)	34.1 (4.4, 63.9)
Conduct	25.0 (6.1, 57.7)	15.4 (1.8, 41.6)
Suicide risk	36.2 (11.9, 59.3)	26.3 (1.5, 57.4)
SUD	35.6 (13.4, 58)	27.9 (4.6, 48.0)
Functional Domain		
Family	0.41 (0, 1.1)	0.34 (0, 1.1)
Work	0.44 (0, 1.2)	0.33 (0, 1.7)
School	0.46 (0, 1.1)	0.39 (0, 1.0)
Life	0.52 (0, 1.6)	0.41 (0, 1.6)
Concept	0.93 (0, 2.4)	0.75 (0, 2.8)
Social	0.29 (0, 1.4)	0.14 (0, 0.7)
Risk	0.05 (0, 0.4)	0.06 (0, 0.6)
Overall	0.43 (0, 1.0)	0.34 (0, 1.1)

No results were statistically significant at the *p* < 0.05 level.

**Figure A1:**
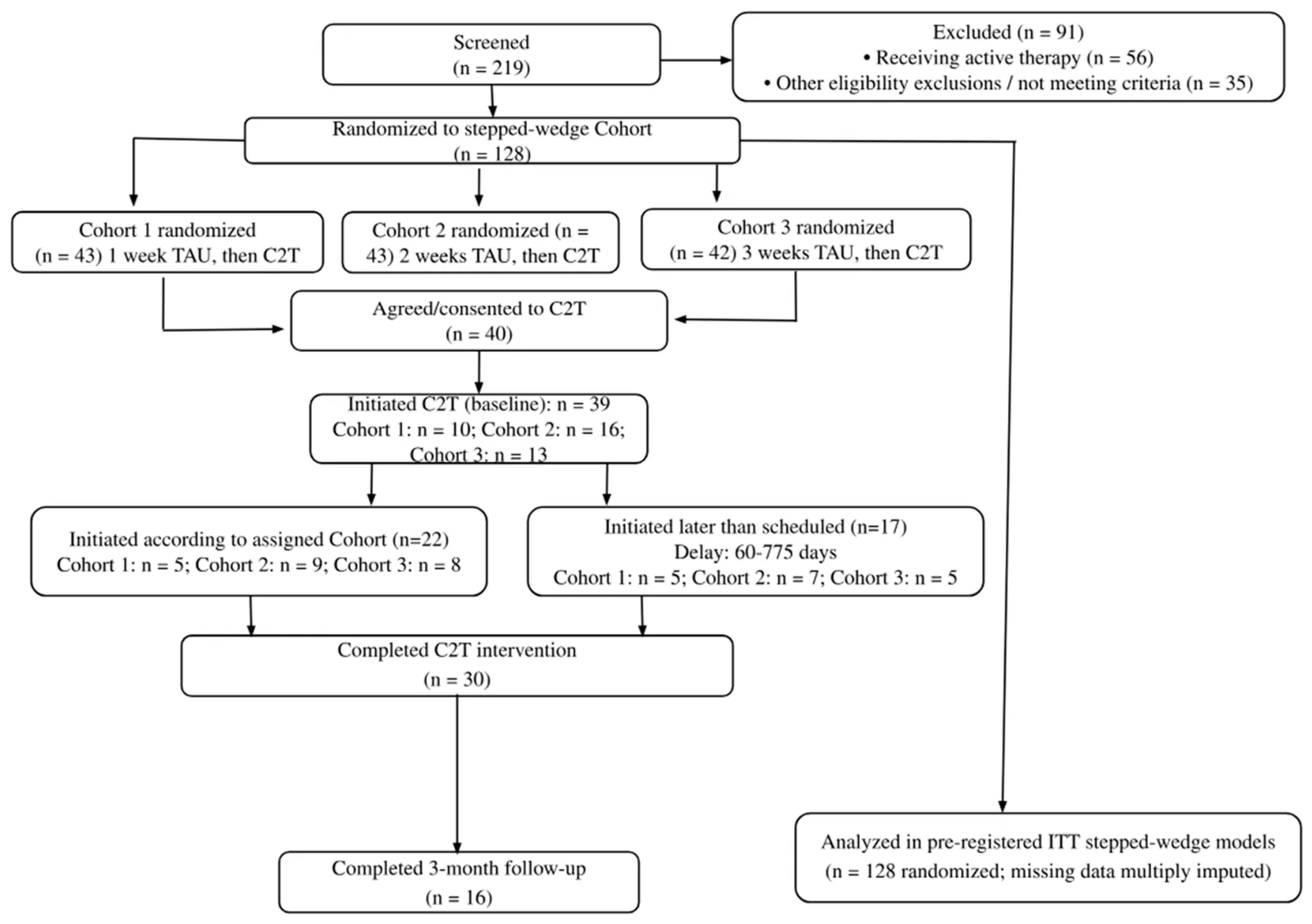
CONSORT Diagram. TAU = treatment as usual; C2T = COPE2Thrive; ITT = intent-to-treat.

## Data Availability

Individual de-identified data, including the data dictionary and statistical code used for analysis, will be made available upon request to the corresponding author, subject to approval by the Cambridge Health Alliance Institutional Review Board and execution of a data use agreement.

## Abbreviations

The following abbreviations are used in this manuscript:

ADHD	Attention Deficit Hyperactivity Disorder
ACT	Acceptance and commitment therapy
AUC	Area Under the Curve
CBT	Cognitive behavioral therapy
C2T	COPE2Thrive
CDC	Centers for Disease Control and Prevention
CI	Confidence interval
COPE	Creating Opportunities for Personal Empowerment
DMHI	Digital mental health intervention
ES	Effect size
ESToRY	Early Screening and Treatment of at-Risk Youth
ITT	Intent-to-treat
K-CAT	Kiddie-Computerized Adaptive Testing
K-SADS	Kiddie Schedule for Affective Disorders and Schizophrenia
MVN	Multivariate normal
NCT	National Clinical Trial
ODD	Oppositional Defiant Disorder
QR	Quick Response
RCT	Randomized controlled trial
REL	Racial, ethnic, and language
SUD	Substance use disorder
TAU	Treatment as usual
WFIRS	Weiss Functional Impairment Rating Scale
WFIRS-S	Weiss Functional Impairment Rating Scale—Self Report
YMHC	Youth mental health crisis
